# A general swimming response in exhausted obligate swimming fish

**DOI:** 10.1098/rsos.211869

**Published:** 2022-09-21

**Authors:** G. Iosilevskii, J. D. Kong, C. G. Meyer, Y. Y. Watanabe, Y. P. Papastamatiou, M. A. Royer, I. Nakamura, K. Sato, T. K. Doyle, L. Harman, J. D. R. Houghton, A. Barnett, J. M. Semmens, N. Ó. Maoiléidigh, A. Drumm, R. O'Neill, D. M. Coffey, N. L. Payne

**Affiliations:** ^1^ Department of Aerospace Engineering, Technion Haifa, 32000 Israel; ^2^ School of Natural Sciences, Trinity College Dublin, Dublin, Ireland; ^3^ Hawaii Institute of Marine Biology, University of Hawaii, Kaneohe, HI 96744, USA; ^4^ National Institute of Polar Research, Tachikawa, Japan; ^5^ Biological Sciences, Florida International University, Miami, FL 33180, USA; ^6^ Organization for Marine Science and Technology, Nagasaki University, Nagasaki, Nagasaki 851-2213, Japan; ^7^ International Coastal Research Center, Atmosphere and Ocean Research Institute, University of Tokyo, Iwate, Japan; ^8^ Zoology, Ecology and Plant Science, Environmental Research Institute, University College Cork, Cork T23 XE10, Ireland; ^9^ Biological Sciences, Queen's University Belfast, Belfast, County Antrim BT9 7BL, UK; ^10^ James Cook University, Cairns, Queensland, Australia; ^11^ Institute of Marine and Antarctic Studies, University of Tasmania, Hobart, Tasmania 7001, Australia; ^12^ Marine Institute, Newport, County Mayo, Ireland

**Keywords:** biologging, elasmobranch, energetics

## Abstract

Marine organisms normally swim at elevated speeds relative to cruising speeds only during strenuous activity, such as predation or escape. We measured swimming speeds of 29 ram ventilating sharks from 10 species and of three Atlantic bluefin tunas immediately after exhaustive exercise (fighting a capture by hook-and-line) and unexpectedly found all individuals exhibited a uniform mechanical response, with swimming speed initially two times higher than the cruising speeds reached approximately 6 h later. We hypothesized that elevated swimming behaviour is a means to increase energetic demand and drive the removal of lactate accumulated during capture via oxidation. To explore this hypothesis, we estimated the mechanical work that must have been spent by an animal to elevate its swim speed and then showed that the amount of lactate that could have been oxidized to fuel it comprises a significant portion of the amount of lactate normally observed in fishes after exhaustive exercise. An estimate for the full energetic cost of the catch-and-release event ensued.

## 1. Introduction

**Figure 1.**
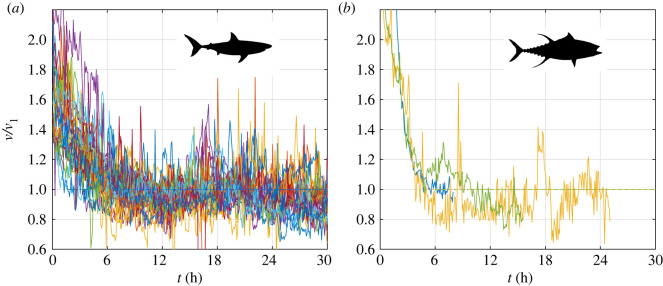
Declining swim speeds (block averaged over 5 min intervals) during the first 24 h after release of 29 individuals from 10 species of sharks and three individuals of Atlantic bluefin tuna. Colours mark different individuals. The ordinate represents instantaneous speed (*v*) as a proportion of the ultimate cruising speed (reached approx. 6 h after release; *v*_1_). Individual traces can be seen in the electronic supplementary material, figures S1, S3 and S6 in Supplementary 1.

Large obligate swimming fish (or ram ventilators), such as many large pelagic species, routinely encounter capture from recreational sports fishers (catch-and-release angling), as bycatch in global longline fisheries (e.g. protected elasmobranchs), or as part of management or research programmes [1,2]. In the field study of large free-ranging fishes, capture and release is required to attach animal-borne bio-loggers, which can directly measure swimming speed [3–7]. Because the capture of these fishes is a stressful event that may last for tens of minutes to several hours, the first 6–9 h after release are well recognized as a ‘stress response’ and commonly excluded from ecological studies (e.g. [3–6]). Looking at these commonly discarded data, we observed an unexpectedly consistent pattern of elevated swimming speeds immediately after release. All animals initially swam almost two times faster than their routine cruising speed; the measured swimming speed gradually decreasing from the time of release to a steady speed we interpret as their routine cruising speed approximately 6 h later (figure 1 and table 1; electronic supplementary material, figure S1 in Supplementary 1). The nature and consistency of this behaviour across many individuals from numerous species, representing a broad range of sizes and habitats, combined with observations that many individuals resumed a variety of regular behaviours during the period of swimming at an elevated speed (electronic supplementary material, figures S3 and S4 in Supplementary 1), led us to reconsider the underlying biochemical and physiological motivations for obligate swimming fish to swim at elevated speeds after strenuous activity.

**Table 1.** Deployment data and key estimates of the energetics of swimming. Full names of the species can be found in the electronic supplementary material, table S1 Supplementary 2. *l* is the total length and *T* is the average water temperature. All other parameters were defined in the text. Means and s.d. are shown for each column.

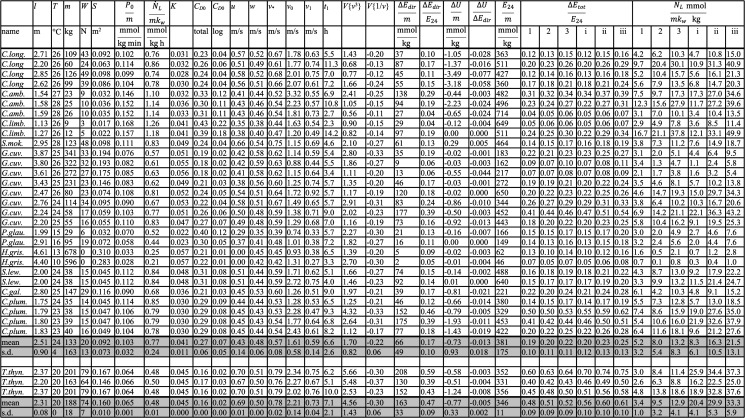

The removal of lactate is an important component of post-exercise recovery. Fighting the hook during capture depletes glycogen stores and generates lactate [8,9], possibly leading to acidosis [10,11]. In turn, post-release recovery replenishes glycogen stores, removes lactate and restores homeostasis [10,12,13]. There are three possible ways by which lactate can be removed: it can be oxidized to pyruvate (either *in situ* or *ex situ*) and re-used in the TCA cycle; it can be converted *in situ* to glycogen via pyruvate kinase reversal [14], or it can be converted *ex situ* to glucose to be reused by all tissues [12,15]. All three ways need oxygen—the first one directly, the others indirectly, through an increase in ATP turnover; the oxygen consumption being linked with ATP production. None of the three ways was conclusively shown to dominate the recovery either in teleosts or in elasmobranchs under controlled laboratory conditions [16–18]. Notwithstanding, swimming at an elevated speed can facilitate each one of them not only because it can increase the oxygen uptake—and hence secure the ATP production—but also because it increases the active metabolic rate and hence increases the rate at which lactate can be oxidized. In fact, the oxidation rate of lactate is inherently limited by the active metabolic rate because the production rate of ATP cannot exceed the rate at which it is consumed.

It is possible that the ability to increase the active metabolic rate is the key to recovery of exhausted fish in general and ram ventilators in particular. Restraining the fish during recovery severely limits its active metabolic rate—and hence limits the rate at which lactate can be oxidized—and undoubtedly affects the way lactate is eventually removed [19]. In fact, neither *in situ* lactate oxidation nor gluconeogenesis were shown to occur at an appreciable rate during recovery of stationary dogfish (*Squalus acanthias*) [16], whereas all lactate was likely to be oxidized during the first hour of recovery of unrestrained salmon [20].^1^ So, perhaps, given sufficient oxygen supply, the animal can use lactate as the energy source for elevated swimming and in this way remove it from its system.

We hypothesize that elevated swimming speeds of ram ventilators collated for this study (figure 1), as well as increased activity of those reported by [20] and decreasing whole-body acceleration in [21], is a manifestation of lactate removal from their system via oxidation. Having only data that was not originally designed to test this hypothesis, we cannot prove it—we can, however, render it plausible by demonstrating that the excess energy needed to swim at an elevated speed could have been provided by oxidation of lactate if its concentration after capture was within the limits found in the literature. We can also show how much more lactate could have been oxidized by elevating the swimming speed compared with an animal swimming at their routine cruising speed over the same time course, or with an animal that is kept stationary. Moreover, by assuming all lactate that could have been oxidized when swimming at the elevated speed was generated during capture, we provide a lower bound for the total energetic cost of a catch-and-release event.

## 2. Methods

### 2.1. Fish tagging

We collated speed data from 29 individual animals of 10 species of sharks and three individuals of Atlantic bluefin tuna (*Thunnus thynnus*). These data were compiled from a number of other research programs [3–6], mostly in the field of ecology. Fish were captured by hook-and-line (drum lines or longlines for sharks and rod and reel for tuna), their total length recorded, and bio-loggers were fitted to their dorsal or pectoral fins using established methods [4]. Being irrelevant to the aim of the respective studies, duration of capture was not recorded for any of the animals—but, in general, most individuals were hooked between 15 and 120 min. All animals remained fully submerged during capture and handling except for the pair of blue sharks (*Prionace glauca*) and three Atlantic bluefin tunas, which were lifted onto the boat deck and instrumented while a hose flushed seawater over their gills. Tagging procedures lasted between 5 and 30 min, but usually less than 10 min. Once instrumented, fish were returned to their environment. Further details on capture and handling can be found in [4] and [6]. All bio-logging devices (Little Leonardo Co., Tokyo, Japan) recorded speed, depth and water temperature every second with accuracy of 0.02 m s^−1^ or higher. The duration of the records varied between 9 and 207 h (electronic supplementary material, table S2 in Supplementary 2).

### 2.2. Direct energetic cost of swimming at an elevated speed

With all animals, the swim speed *v* started at the initial speed *v*_0_ and reached what we interpret as a steady value *v*_1_ after time *t*_1_. The energy used during that time can be found by the quadrature

2.1 $$E\{v;t_{1}\}=\int_{0}^{t_{1}}P(t)\;dt\;$$

of the active metabolic rate,

2.2 $$P(t)=P_{0}+P_{T}(t),$$

which naturally splits into the standard metabolic rate *P*_0_, and the metabolic rate needed to power the animal through water

2.3 $$P_{T}(t)=\frac{T(t)v(t)}{\eta \eta_{m}},$$

where *T*(*t*) is the thrust, *η* is the hydrodynamic propulsion efficiency and *η_m_* is the chemo-mechanical efficiency of the locomotive muscles (following [22], we have used 0.7 and 0.8 for the two, respectively). The thrust

2.4 $$T(t)=D(t)+\frac{d}{dt}U(t),$$

acts both to overcome the hydrodynamic resistance

2.5 $$D(t)=\frac{1}{2}\rho SC_{D0}v^{2}(t)+\frac{2KL^{2}(t)}{\rho Sv^{2}(t)},$$

and to increase the mechanical energy,

2.6 $$U(t)=-Wd(t)+\frac{1}{2}mv^{2}(t),$$

of the swimmer. In (2.5), *ρ* is the density of water, *S* is the reference area, chosen here as the maximal cross-section area of the body in the transverse plane, *C_D_*_0_ and *K* are a pair of hydrodynamic coefficients (associated with the parasite and induced constituents of drag)^2^ and *L* is the hydrodynamic lift. For most species on our list, *C_D_*_,0_ ∼ 0.3 and *K* ∼ 0.04 with relatively small variance (table 1; §2.9). In (2.6), *d* is the swimming depth, *m* is the body mass and *W* is the submerged weight.^3^ It can be expressed as

2.7 W=βgm,

where *g* is the acceleration of gravity and, by interpretation, *β* is the ratio between the submerged and out-of-water weights of the animal (akin to the sinking factor). The reader is referred to [22] for details. For most sharks, *β* ranges between a few negative thousandths [23] and a few positive hundredths [24].

To simplify the following analysis, it will be assumed that most of the time the animal swims at small angles relative to the horizon, rendering the hydrodynamic lift to be approximately equal to the submerged weight,

2.8 L(t)≈W.

In this case, the drag becomes a function of speed only, and it can be expressed as

2.9 $$D(t)\approx \frac{1}{2}\rho SC_{D0}\left(v^{2}(t)+\frac{u^{4}}{v^{2}(t)}\right),$$

where

2.10 $$u={\left(\frac{K}{C_{D0}}\right)}^{1/4}{\left(\frac{2W}{\rho S}\right)}^{1/2}$$

is a certain combination of parameters having dimensions of speed.^4^ The reader is referred to [22] for details.

Collecting it all together, the energy that the animal spent from the point of release and until time *t*_1_ is the sum

2.11 $$E\{v;t_{1}\}=P_{0}t_{1}+E_{D}\{v;t_{1}\}+\frac{U(t_{1})}{\eta \eta_{m}}$$

of the energy needed to supply the standard metabolic needs, *P*_0_*t*_1_, the energy needed to overcome the hydrodynamic resistance

2.12 $$E_{D}\{v;t_{1}\}\approx \frac{\rho SC_{D0}}{2\eta \eta_{m}}\left(\int_{0}^{t_{1}}v^{3}(t)\;dt\;+u^{4}\int_{0}^{t_{1}}\;\frac{1}{v(t)}dt\right),$$

and the (typically negative) correction, *U*(*t*_1_)/*ηη_m_*, due to a change in the total mechanical energy;

2.13 $$P(t)\approx P_{0}+\frac{\rho SC_{D0}}{2\eta \eta_{m}}\left(v^{3}(t)+\frac{u^{4}}{v(t)}\right)+\frac{1}{\eta \eta_{m}}\frac{dU(t)}{dt}$$

is its active metabolic rate. If the animal was swimming at constant speed *v*_1_ = *v*(*t*_1_) and constant depth *d*_1_ = *d*(*t*_1_) all the time, the energy used up to *t*_1_ would have been

2.14 $$E\{v_{1};t_{1}\}=P_{0}t_{1}+E_{D}\{v_{1};t_{1}\},$$

where

2.15 $$E_{D}\{v_{1};t_{1}\}=\frac{\rho SC_{D0}v_{1}^{3}t_{1}}{2\eta \eta_{m}}\left(1+\frac{u^{4}}{v_{1}^{4}}\right).$$

The ubiquitous factor outside the parentheses, which has the dimensions of energy, will be denoted *E*_1_ below. It can be reduced to

2.16 $$E_{1}=\frac{\rho SC_{D0}v_{1}^{3}t_{1}}{2\eta \eta_{m}}=\frac{C_{D0}}{\eta \eta_{m}k_{m}}\frac{mv_{1}^{2}}{2}\frac{v_{1}t_{1}}{l}$$

by using the mass *m*, the (fork) length *l*, and the prismatic coefficient *k_m_*, which is the ratio of the volume of the body (with no fins) and the volume of the minimal cylinder enclosing it [22].

The direct energetic cost of swimming at an elevated speed is the difference

2.17 $$\Delta E_{dir}\{v;t_{1}\}=E\{v;t_{1}\}-E\{v_{1};t_{1}\}$$

between (2.11) and (2.14). It can be recast as

2.18 $$\Delta E_{dir}\{v;t_{1}\}=E_{1}\left(V\{v^{3};t_{1}\}+\frac{u^{4}}{v_{1}^{4}}V\{v^{-1};t_{1}\}\right)+\frac{1}{\eta \eta_{m}}\left(-Wd_{1}+\frac{1}{2}mv_{1}^{2}\right),$$

where with any strictly positive integrable function *f*,

2.19 $$V\{f;t_{1}\}=\frac{1}{t_{1}}\int_{0}^{t_{1}}\left(\frac{\;f(t)}{\;f(t_{1})}-1\right)\;\;dt.$$

For all animals on our list, the second term on the right of (2.18), which manifests the difference in mechanical energy of the animal between the end-points of the swimming interval, turns out to be a few per cent of the first and therefore can be neglected (table 1).

### 2.3. Lactate accumulation and glycogen turnover

Strenuous attempts of an animal to resist capture are fuelled mainly by anaerobic catabolism of glycogen [16,25]. Glycolysis of a single glycogen unit yields three molecules of ATP and two molecules of lactate, which accumulate in the body. Glycogen needs to be replenished after the release, but the cost of its return depends on substrates and pathways used for its synthesis. It takes five ATP molecules to reform two lactate molecules back into a unit of a glycogen chain and seven ATP molecules if free glucose is an intermediate [26]. Two ATP molecules can be saved if glycerol replaces lactate as the substrate, but the viability of this pathway in elasmobranchs is not certain. Lactate needs to be removed after the release, and it can be used either as a substrate for gluconeogenesis, or as a substrate to generate ATP. In fact, oxidation of a single lactate molecule can yield up to 16 molecules of ATP—the same number as would have been obtained if the catabolism of glycogen would not have stopped after glycolysis because of the lack of oxygen [27,28].

Summing it up in a formal way, *N_G_* moles of glucose taken from glycogen reserves during capture supplies

2.20 $$\Delta E_{-G}=3eN_{G}$$

of usable energy, where *e* is an energetic equivalent of ATP, approximately 30 J mmol^−1^—the energy released during hydrolysis of a single ATP molecule into ADP. It also leaves

2.21 $$N_{L}=2N_{G}$$

moles of lactate. If oxidized, they may add

2.22 $$\Delta E_{L}=n_{LO}eN_{L}$$

of usable energy, where *n_LO_* is the number of ATP molecules that can be obtained from oxidation of a single lactate molecule (approx. 16). Excess energy

2.23 $$\Delta E_{+G}=n_{GLU}eN_{G}$$

will be required after the release to replenish the glycogen to its original concentration; *n_GLU_* can be anywhere between 3 and 7, depending on substrates and pathways. To remain conservative in our estimations, we have used *n_GLU_* = 3. It will be shown in §2.6 to be a small fraction of the total energetic cost of a capture-and-release event.

### 2.4. Phosphocreatine turnover

There is an implicit turnover of high-energy substrates other than glycogen, most notably phosphocreatine (PCr). PCr concentrations in white muscles are similar to those of glycogen [16]. However, because its energetic value is equivalent to that of ATP [29], whereas the energetic value of each glycogen unit is a few tens moles of ATP, we deem the effect of PCr turnover on the energy balance negligible. As opposed to the turnover of glycogen, this turnover is quick, both in teleosts [29] and elasmobranchs [16], and the concentration of PCr recovers within a few tens of minutes after the end of exercise. Consequently, PCr was probably the source of energy for high-speed bursts observed in some individuals a few hours after release (electronic supplementary material, figure S5 in Supplementary 1).

### 2.5. Estimating the lactate turnover

We hypothesize that lactate is not used as a source of carbon in gluconeogenesis, which relies on other substrates, but rather is oxidized and fuels, at least in part, the metabolic demands of swimming at an elevated speed. Taking the cue from [20], three scenarios that prescribe that part are considered viable:

(i) Lactate fuels all metabolic needs above the routine metabolic rate—in other words, the oxidation rate of lactate matches the difference between active metabolic rate when swimming at an elevated speed and the active metabolic rate when swimming at *v*_1_.
(ii) Lactate fuels all metabolic needs of the animal above, but not including, the standard metabolic rate—in other words, the oxidation rate of lactate matches the difference between active and standard metabolic rates.
(iii) Lactate fuels all metabolic needs of the animal.

The resulting amounts of consumed lactate can be compared with the amounts of lactate that could have been oxidized in a few reference (hypothetical) scenarios:

(1) Lactate fuels the standard metabolic rate when the animal is not allowed to move.
(2) Lactate fuels all metabolic needs of the animal above, but not including, the standard metabolic rate when swimming at *v*_1_.
(3) Lactate fuels all metabolic needs of the animal when swimming at *v*_1_.

Estimates of consumed lactate can also be converted into the amount of glycogen that must have been consumed during capture to produce them. And if one is ready to accept the hypothesis that no appreciable amount of lactate has been left at *t*_1_, one can convert the estimated amount of glycogen into a lower bound of the energetic cost of the entire capture-and-release event.

Formally, all six scenarios can be represented by a single equation,

2.24 $$\Delta E_{L}\approx s_{123}\Delta E_{dir}\{v;\;\;t_{1}\}+s_{23}E_{D}\{v_{1};\;\;t_{1}\}+s_{3}P_{0}t_{1},$$

where *s*_123_, *s*_23_ and *s*_3_ are switching parameters (taking on the values 0 or 1): *s*_123_ is unity for scenarios (i), (ii) and (iii) and zero for (1), (2) and (3); *s*_23_ is unity for all scenarios except for (i) and 1; *s*_3_ is unity for scenarios (iii), (1) and (3). The amount of lactate that has to be oxidized to provide that energy is Δ*E_L_*/(*n_LO_e*) by (2.22). Its explicit form,

2.25 $$N_{L}=\frac{E_{1}}{n_{LO}e}{\bar{N}}_{L},$$

in which

2.26 $${\bar{N}}_{L}=s_{123}\left(V\{v^{3};\;\;t_{1}\}+\frac{u^{4}}{v_{1}^{4}}V\{v^{-1};\;\;t_{1}\}\right)+s_{23}\left(1+\frac{u^{4}}{v_{1}^{4}}\right)+s_{3}\frac{P_{0}t_{1}}{E_{1}}$$

follows by (2.15), (2.18) and (2.10).

### 2.6. Energetic cost of a catch-and-release event

The amount of glycogen that must have been consumed anaerobically to generate *N_L_* moles of lactate is *N_G_* = *N_L_*/2 by (2.21). If one is ready to assume that all lactate has been oxidized by the time the speed returns to normal, one can now furnish a conservative lower limit

2.27 $$\Delta E_{tot}=\Delta E_{-G}+\Delta E_{+G}+\Delta E_{dir}\{v;\;\;t_{1}\}$$

of the energetic cost of the entire catch-and-release event. It is conservative because not all lactate could have been oxidized up to the time *t*_1_, and because additional cost can be associated with aerobic effort during capture and with turnover of other energetic substances (PCr in particular). It can be put in two equivalent forms. The first one,

2.28 $$\Delta E_{tot}=\left(1+s_{123}\frac{3+n_{GLU}}{2n_{LO}}\right)\Delta E_{dir}\{v;\;\;t_{1}\}+\frac{3+n_{GLU}}{2n_{LO}}(s_{23}E_{D}\{v_{1};\;\;t_{1}\}+s_{3}P_{0}t_{1}),$$

follows (2.27) by (2.20)–(2.24); the second one,

2.29 $$\Delta E_{tot}=E_{1}\Delta {\bar{E}}_{tot},$$

follows the first by (2.18) with

2.30 $$\begin{array}{rl} \Delta {\bar{E}}_{tot}\approx & \left(V\{v^{3};t_{1}\}+\frac{u^{4}}{v_{1}^{4}}V\{v^{-1};t_{1}\}\right)\left(1+s_{123}\frac{3+n_{GLU}}{2n_{LO}}\right)\\ & +\left(s_{23}\left(1+\frac{u^{4}}{v_{1}^{4}}\right)+s_{3}\frac{P_{1}t_{1}}{E_{1}}\right)\frac{3+n_{GLU}}{2n_{LO}}. \end{array}$$

It is noted that the ratio *P*_0_*t*_1_/*E*_1_, found in (2.26) and (2.30), is equivalent to the ratio $2w^{3}/v_{1}^{3}$, where

2.31 $$w={\left(\frac{\eta \eta_{m}P_{0}}{\rho SC_{D0}}\right)}^{1/3}$$

was interpreted in [22] as the speed that minimizes the cost of transport of a neutrally buoyant swimmer.

For all animals on our list, Δ*E_dir_*{*v*; *t*_1_}, *E_D_*{*v*_1_; *t*_1_} and *P*_0_*t*_1_ are comparable quantities (see electronic supplementary material, table S2 in Supplementary 2), whereas (3 + *n_GLU_*)/2*n_LO_* ranges between 1.2 and 1.3, depending on the pathway of gluconeogenesis (see §2.3). Consequently (and perhaps counterintuitively), the energy spent on swimming at an elevated speed, Δ*E_dir_*{*v*; *t*_1_}, comprises the major part of the total energetic cost of the capture-and-release event, Δ*E*_*tot*_.

### 2.7. Standard metabolic rate

The standard metabolic rate can be estimated using an allometric scaling equation

2.32 $$P_{0}=k_{P}m^{\alpha }e^{-k_{\tau }/\tau },$$

where *τ* is the absolute body temperature, whereas *k_P_*, *α* and *k_τ_* are certain phenomenological parameters. Their typical values are 85 mole ATP per seconds per kg*^α^*, 0.8 and 5020 K, respectively [30], but there can be significant interspecific differences in these parameters [30,31]. In particular, *k_P_* can differ almost two-fold. Current mass–metabolic-rate scaling relationships specifically for elasmobranchs suffer from large estimation uncertainty because the few direct measurements were obtained for young or small animals and hence are invariably biased by the metabolic rate of growth [32]. Moreover, there is an intrinsic difficulty of measuring standard metabolic rate for obligate swimmers. The particular value of *k_P_* cited above was recalculated from the one found in [30], by assuming that under normal circumstances a single mole of O_2_ yields approximately 4 moles ATP (see §2.8.3). Having updated the ATP to O_2_ ratio, it is 4/6 of the value used in [22].

### 2.8. Reference quantities

A few reference parameters will be needed in the course of the following discussion. One is the speed that minimizes the cost of transport, *v*_∗_. The other is the (routine) daily energy expenditure, *E*_24_. The third is the standard oxidation rate, ${\dot{N}}_{L,0}$—the oxidation rate of lactate at which the energy released exactly offsets the standard metabolic rate of the animal. The last two are the aerobic scope, *P*_max_—the maximal swimming power that can be sustained aerobically—and the part that the white muscles can take in this effort, $P_{\max}^{\left(w\right)}$.

#### 2.8.1. Routine daily energy expenditure

The routine metabolic rate can be defined in two ways. The first one is consistent with our interpreting the day-averaged speed *v*_1_ as the routine cruising speed. In this case, the routine metabolic rate is given by the variant of (2.13) with *v* = *v*_1_ and d*U*(*t*)/d*t* = 0, and the routine daily energy expenditure,

2.33 $$E_{24}=E\{v_{1};t_{24}\}=P_{0}t_{24}+E_{D}\{v_{1};t_{24}\},$$

is its product with the duration of the day *t*_24_; actually, it is a variant of (2.14) with *t*_24_ replacing *t*_1_. It takes on the explicit form

2.34 $$E_{24}=E_{1}\frac{t_{24}}{t_{1}}\left(1+\frac{u^{4}}{v_{1}^{4}}+\frac{P_{0}t_{1}}{E_{1}}\right)=E_{1}\frac{t_{24}}{t_{1}}\left(1+\frac{u^{4}}{v_{1}^{4}}+2\frac{w^{3}}{v_{1}^{3}}\right)$$

by (2.15), (2.10) and (2.31).

The second option is to adopt the hypothesis that a predator swims, on average, so as to maximize the difference between the energy gained from prey and the energy spent in search of it. In most cases, this hypothesis implies swimming near the speed that minimizes the cost of transport, *v*_∗_. The routine energy expenditure in this case will be given by the variant

2.35 $$E_{24*}=E_{1}\frac{t_{24}}{t_{1}}\left(1+\frac{u^{4}}{v_{*}^{4}}+2\frac{w^{3}}{v_{*}^{3}}\right)$$

of (2.34) with *v*_∗_ instead of *v*_1_. Following [22], this optimal speed can be approximated with

2.36 $$v_{*}\approx w\left(1+\frac{3}{14}\frac{u^{3}}{w^{3}}\right);$$

the reader is referred to Table 2 ibid. Under this hypothesis, a match between *v*_1_ and *v*_∗_ can serve to test the coherence of our estimates.

#### 2.8.2. Unit oxidation rate

It was mentioned already that rate ${\dot{N}}_{L}$ at which lactate can be oxidized is limited because ATP production cannot exceed its demand. When an animal does not move and no gluconeogenesis takes place, its active metabolic rate tends to the standard one, and in this case, the maximal oxidation rate of lactate cannot exceed

2.37 $${\dot{N}}_{L,0}=P_{0}/\left(n_{LO}e\right).$$

Essentially, this is scenario 1 mentioned earlier: equation (2.37) follows (2.25) and (2.26) with *s*_123_ = *s*_23_ = 0 and *s*_3_ = 1. ${\dot{N}}_{L,0}$ will be referred to as the ‘unit oxidation rate’ and serve a reference in discussion.

#### 2.8.3. Aerobic scope

Reported maximal rate of ATP production by oxidative phosphorylation in salmonids is 29 mmol per min per kg red muscle [33]—it will be denoted ${\dot{\bar{N}}}_{15}^{\left(r\right)}$—and 3.5 mmol per min per kg white muscle [34]—it will be denoted ${\dot{\bar{N}}}_{15}^{\left(w\right)}$. These values were converted from oxygen consumption measured in isolated mitochondria at 15°C, and were based on the assumption that 6 moles ATP are generated for each mole of O_2_ used. We are not aware of comparable studies on elasmobranchs. Acknowledging the potential for error between *in vitro* and *in vivo* rates, and a potential for different mitochondrial densities and compositions, we cautiously adopt these figures here. To this end, however, they need to be corrected for temperature (see below), and they need to be adjusted for a different ATP to O_2_ ratio. This ratio is currently believed not to exceed 5.1 moles ATP for each mole of O_2_, even with as few as 8 c-units in the ring of the F-ATP complex [28]. Cautiously reducing this number to 4 moles ATP for each O_2_ to account for non-ideal mitochondrial efficiency, we proceed with maximal rates of 19.3 and 2.3 mmol ATP per min per kg red and white muscles, respectively.

Temperature correction was suggested in [34] as a factor

2.38 $$f(\tau )\approx Q_{10}^{(\tau -\tau_{0})/10}$$

with ${\dot{\bar{N}}}_{15}^{\left(r\right)}$ and ${\dot{\bar{N}}}_{15}^{\left(w\right)}$, where *τ*_0_ = 288K and *Q*_10_ ≈ 1.5. Consequently,

2.39 $$P_{\max}(\tau )=mef(\tau )(k_{w}{\dot{\bar{N}}}_{15}^{\left(w\right)}+k_{r}{\dot{\bar{N}}}_{15}^{\left(r\right)})$$

and

2.40 $$P_{\max}^{\left(w\right)}(\tau )=mek_{w}f(\tau ){\dot{\bar{N}}}_{15}^{\left(w\right)},$$

where *k_w_* and *k_r_* are mass fractions of white and red muscles, respectively. Following [35,36], we assume that white muscles comprise 50% of the body mass (*k_w_* = 0.5), whereas red muscles comprise 3% (*k_r_* = 0.03) in elasmobranchs and 7% in tuna. We acknowledge that these fractions can significantly vary between different species and even between different individuals of the same species. In fact, we will use *k_r_* = 0.07 with tuna [36].

### 2.9. Application of theory to our data

Mass was estimated from measured length using mass-length regressions from FishBase [37]. Fins' dimensions were estimated from measured length based on known length ratios. Those of *C. limbatus, C. plumbeus*, *G. cuvier* and *P. glauca* was taken from [22]; those of *C. longimanus* was taken from [5]. We have used the mid-range values cited in both references. Length ratios of *S. mokarran* were taken from [3], and *S. lewini* was assumed morphologically similar to *S. mokarran*. *C. galapagensis* and *C*. *amblyrhynchos* were assumed morphologically similar to *C. obscurus*, for which the relevant data can be found in [22]. Length ratios of *H. griseus* and of *T. thynnus* were taken as average values from a few photographs. All fins of tuna were assumed extended during swimming. Hydrodynamic coefficients were estimated using the method described in the electronic supplementary materials of [22] and [5].^5^ The reference Reynolds number for drag estimations was based on swimming speed of 0.7 m s^−1^ for all individuals but the two *H. griseus*, for which 0.3 m s^−1^ was used. Parasite drag coefficient *C_D_*_0_ was corrected for the resistance of the gills (by increasing the body drag by 20%) and for the drag of the data logger, which was assumed to add a constant 38 cm^2^ to the drag area *SC_D_*_0_ (see Supplementary of [5]). For small animals on our list (*C*. *limbatus*), the addition of the data logger doubled the drag coefficient (see electronic supplementary material, table S2).

The missing density ratios of the animals were taken from the same references as the missing morphological parameters. *H. griseus* was assumed neutrally buoyant after [23] (where it was found very slightly positively buoyant). All density ratios were adjusted to within one-hundredth of the original estimates so as to keep the power during the initial dive non-negative. Tunas have a swim bladder and hence their density ratio was adjusted toward the end of the initial dive. All estimates can be found in table 1 and in the electronic supplementary material, table S2.

The routine swimming speed *v*_1_ was defined as the average speed during 24 h (or the length of the record) following the first 6 h; averaging over 24 h smooths out the diel-cycle variations of swimming speed. The recovery time *t*_1_ was defined as the time at which the speed dropped below *v*_1_ for 20 consecutive minutes (figure 1), *V*{*v*^3^; *t*_1_} and *V*{*v*^−1^; *t*_1_} were estimated with (2.19) using simple trapezoidal integration. The sink rate and the acceleration, which are inseparable parts of d*U*/d*t*, were found by differentiating a running 21-point parabolic fit of depth and speed, respectively. *P*_0_ was estimated with (2.32) based on average water temperature. *P*_max_/*m* and $P_{\max}^{\left(w\right)}/m$ were estimated with (2.38), (2.39) and (2.40) based on average water temperature and mass fractions cited in the paragraph preceding (2.39). Sensitivity of our results to assumed parameters is addressed in §4.

## 3. Results

In general, the swimming speed gradually decreased after release to what we interpret as a regular cruising speed, which was reached approximately 6 h after release (all species, 6.6, s.d. 2.5 h) (figure 1 and table 1; electronic supplementary material, figure S1 in Supplementary 1). This speed (all species, 0.60, s.d. 0.14 m s^−1^) was similar to the cost of transport (COT) optimal speed *v*_∗_ predicted by (2.36) (all species, 0.59, s.d. 0.1 m s^−1^); in most cases, the particular differences between the two fall within our estimation error (table 1). Toward the end of that period, some of the animals resumed ‘yo-yo’ diving (electronic supplementary material, figures S2 and S4 in Supplementary 1). These animals were *C*. *longimanus* (#1 and #2), *S. lewini* (#2) and most of the tiger sharks (*Galeocerdo cuvier*). Some other animals made short high-speed bursts (electronic supplementary material, figures S3 and S5 in Supplementary 1). These animals were *C*. *longimanus* (#2 and #4), *C. galapagensis*, *P. glauca* (#2), *S. lewini* (#2) and some of the tiger sharks. Not on this list, but some tagged sharks were recaptured on a hook in less than 1 h after their release (authors, personal observation). Except for those short bursts, the swimming speed during that period was within the aerobic scope of the animals (electronic supplementary material, figures S5 and S8 in Supplementary 1); moreover, it was within the aerobic scope of the white muscles.

Practically all animals dove with no power immediately after their release. These initial dives were used to verify the sinking factor; in fact, the power needed to move them through the water (*P_T_*) cannot be negative because it is impossible to extract metabolic power from mechanical (electronic supplementary material, figure S8 in Supplementary 1). The sinking factor of tunas, which have a swim bladder, was verified by the end of the dive.

**Figure 2.**
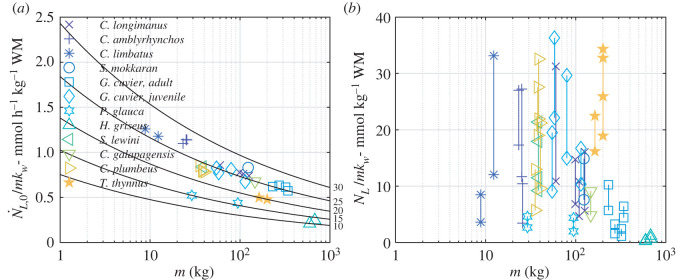
(*a*) Unit oxidation rate of lactate (equation (2.37)) as a function of body mass and temperature (indicated next to the respective line in °C). The rate is reduced by the mass of white muscles. A 4.5°C increase in temperature increases the unit oxidation rate by 20%. Points mark the individual animals, colour- and symbol-coded by species. (*b*) The amount of lactate (per unit mass of the white muscles) that could have been oxidized to fuel the swimming at the elevated speed (scenarios (i) and (ii)). Vertical lines connect the estimate when lactate fuels the difference between swimming at an elevated speed and swimming at *v*_1_ (lower point; this is scenario (i)) and the estimate when it fuels all energetic costs above the standard metabolic rate (higher point, scenario (ii)).

For all animals on our list, the unit oxidation rate of lactate was estimated with equation (2.37) to be less than 1.2 mmol h^−1^ kg^−1^ (mean 0.77; s.d. 0.24), decreasing with mass and increasing with body temperature (figure 2). Since maximal lactate concentration in elasmobranchs muscles can reach 40 mmol kg^−1^ (e.g. [16]), this result implies that it can take a large ectothermic animal more than 40 h to oxidize its lactate at the standard metabolic rate, even if its tissues were capable of readjusting to lactate as a metabolic fuel on the time scale of a few hours.

Swimming at an elevated speed allows for oxidizing up to 22 mmol lactate per kg white muscle (elasmobranchs, 8.3, s.d. 6.1; tunas, 20.4, s.d. 4.1) if oxidation of lactate fuels the energy needed to swim at an elevated speed (scenario (i), lower points on figure 2*b*), and up to 36 mmol lactate per kg white muscle (elasmobranchs, 16.3, s.d. 10.5; tunas 29.9, s.d. 5.3) if it fuels all energetic needs above the standard metabolic rate (scenario (ii), upper points on figure 2*b*). The smallest estimates are for large animals residing in cold water (*H. griseus*, 0.3 and 0.7 mmol per kg in scenario (i), 0.4 and 1.2 mmol per kg in scenario (ii)); the largest estimates are for a warm-water small-to-medium-sized elasmobranchs (*C. amblyrhyncos,* juvenile *G. cuvier, C. longimanus, C. limbatus* and *C. plumbeus*). The amount of lactate that could have been oxidized at the standard metabolic rate (scenario (1)) would have been less than 17 mmol kg^−1^ (elasmobranchs, 5.2, s.d. 3.2; tunas 3.4, s.d. 1.0; the few large numbers correlate with exceptionally long recovery periods).

Direct energetic cost of swimming at an elevated speed comprised up to 39% of the estimated daily energy expenditure (elasmobranchs, 17, s.d. 10; tunas 47, s.d. 9). Considering that this energy came from lactate, and that the lactate was generated anaerobically from glycogen, and that the glycogen needs to be restored (this is scenario (i)), this estimate rises up to 47% (elasmobranchs, 20, s.d. 12; tunas 56, s.d. 10). Scenario (ii) adds 3 to 5% (absolute) to these estimates.

Referring to equation (2.18), the change in mechanical energy *U* (this is the last term on the right of this equation) between the point of release and time *t*_1_ comprised less than 8% (all species, 1.3, s.d. 1.8) of the energy spent on swimming at an elevated speed. It furnishes an *a posteriori* justification for the neglect of this term in the analysis.

## 4. Discussion

In data we would typically discard in an ecological study, we found an unexpected general pattern of elevated swimming speeds in exhausted fish and were curious as to why fish would swim faster than cruising speeds for prolonged durations (figure 1). A similar trend of decreasing whole-body acceleration after capture and release was reported in [21]. Taking a biochemical and mechanical modelling perspective, we hypothesized that this behaviour reflects the physiological state of the animals and the tight relationship between their ventilation and metabolic rate. Distinguishing the mode of ventilation is important as facultative versus obligate swimming imposes different physiological constraints on metabolism. An obligate swimming fish can obtain oxygen for aerobic respiration after release but may not while stationary, whereas facultative swimmers, like many teleosts, can respire using active pumping. That said, neither can significantly increase their active metabolic rate without moving. As maximal oxidation rate of lactate is coupled to active metabolic rate, when an animal does not move (for example, by being restrained), its active metabolic rate is close to standard metabolic rate and maximal oxidation rate is remarkably small. This is particularly true for large ectothermic animals in cold water (figure 2*a*). With no option to move, quicker removal of lactate can be achieved only by its reuse as a source of carbon in endogenous or exogenous gluconeogenesis. This conclusion is in accordance with the results shown in dogfish [16] and in skipjack tuna [12]. Indeed, the last study estimated that tuna are unable to oxidize lactate when stationary (and anaesthetized) despite their high aerobic capacity and hypothesized that free swimming may influence the rate of lactate clearance. A moving animal increases its active metabolic rate allowing for lactate to be oxidized at a higher rate, as, in fact, demonstrated in salmonids (e.g. [20,38]). All animals on our list increased their active metabolic rate many-fold over their routine metabolic rate immediately after release (electronic supplementary material, figures S5 and S8 in Supplementary 1), and opened up the possibility to oxidize lactate—either *in situ* (i.e. in white muscles) or in the neighbouring red muscles [39].

Animals on our list swam within the aerobic scope of white muscles, suggesting that lactate could, in principle, be oxidized *in situ*, within the white muscles. This observation renders both scenarios (i) (in which all metabolic needs above the routine metabolic rate are met by oxidation of lactate) and (ii) (in which all metabolic needs above the standard metabolic rate are met by oxidation of lactate) theoretically possible. By scenario (ii), all the animals on our list could have oxidized, on average, almost half of the lactate that would have been accumulated in their muscles should they have fought the hook to exhaustion (approx. 40 mmol per kg muscle [16]). Elevating the swim speed (above *v*_1_) contributed about half of this estimate—this number can be inferred from comparing scenarios (i) and (ii).

Our conclusions about the possibility of lactate oxidation do not exclude the possibility of other responses to capture occurring. We do not know for certain what drove the animals to swim at an elevated speed, and what caused them to stop swimming at the elevated speed. Several behavioural or ecological motivations could contribute towards why these fish swam at an elevated speed, but these reasons do not exclude the fact that elevated swimming still serves a purpose to remove lactate from the system after capture and release. For example, the short high-speed bursts in excess of their aerobic scope could be interpreted as a flight response powered in addition to lactate oxidation. Thus said, the exponential-like decay on figure 1*a* hints toward Michaelis-Menten-type kinetics, where the reaction rate diminishes with diminishing amount of a substrate—the lactate in our case. Should this be the case, the return to normal swim speed should indicate a complete removal of lactate from the system, and since the estimated amount of lactate did not exceed 40 mmol kg^−1^ muscle in any scenario, it implies that either not all individuals had fought to exhaustion or that the maximal lactate concentration in muscles differs among species and among individuals. Regardless of how much lactate is produced during capture, we find that elevating the swim speed can significantly expedite its removal from the system.

Based on scenario (ii) (and on the assumption that no lactate was left in the system after the swimming speed has returned to normal), the total energetic cost of capture-and-release is, on average, slightly less than 25% of the daily energy expenditure, but can exceed 70% with tuna. Notably, it can be a large underestimate for a tuna, which can use its large aerobic scope to resist capture or power burst-swimming [40]. We have no means to estimate the aerobically consumed energy during capture in natural settings for the animals considered here.

We find that repeating the analysis with different parameter values do not change our conclusions. For example, increasing the ATP to O_2_ ratio by 25%, from 4 to 5, is equivalent to raising the temperature in figure 2*a* by 5°C, and hence lactate removal at standard metabolic rate will still take tens of hours; the amount of oxidizable lactate shown in figure 2*b* remains unchanged. It raises the baseline of the estimated power requirement for swimming (as shown in the electronic supplementary material, figures S5 and S8), but it also raises the aerobic scope of the muscles, meaning the conclusion that the animal swam within the aerobic capacity of their white muscles is also unchanged. As mentioned already at the end of §2.6, the energetic cost of the entire catch-and-release event is practically independent of the basic metabolic rate and is, essentially, the energy spent on swimming at the elevated speed. A comparison between electronic supplementary material, tables S2 and S3, in Supplementary 2 attests to this conjecture.

The amount of lactate (per mass *mk_w_* of the white muscles) used to fuel the swimming at the elevated speed scales with *E*_1_/(*mk_w_*)—see equation (2.25). In turn, *E*_1_/(*mk_w_*) depends on the combination $v_{1}^{2}(v_{1}t_{1}/l)$ of directly measured parameters and a combination *C_D_*_0_/*ηη_m_k_m_k_w_* of guessed or estimated ones—see equation (2.16). Any change in this factor should proportionally change the estimate of the amount of oxidized lactate. We do not envision a sufficiently large error in this factor would affect our conclusions.

Significant challenges underpin the study of large free-ranging obligate swimming fishes because pelagic animals are inaccessible relative to freshwater animals, and large animals are more challenging to hold in aquaria for study than small ones. Bio-loggers are one solution to overcome the challenges of field study for such animals [41]. Our study demonstrates how combining theoretical modelling approaches with swimming speed data we would normally exclude adds value to these hard-to-collect data and gain insight into the behaviour and physiology of these hard-to-study animals that routinely encounter catch-and-release angling. Thus said, currently used bio-loggers are inapplicable to small animals. The drag area (the product of drag coefficient and its reference area) of a current bio-logger approximately equals the area of its transverse cross-section. The drag area was estimated at 38 cm^2^ for all bio-loggers used to collect this dataset. At the same time, the drag area of a fusiform shark is, approximately, one-fifth of the area of its transverse section (see electronic supplementary material, table S2 in Supplementary 2). Attaching a bio-logger can represent a significant increase in total drag to an animal because the drag areas for the animal and the bio-logger are additive. For example, attaching a bio-logger doubles the drag for a 1.2 m *C. limbatus* that has a drag area of 40 cm^2^*.* Thus, the behaviour of an animal after attaching a bio-logger cannot be considered ‘normal’ even after its speed has been stabilized.

## Data Availability

The raw data underlying the study can be found on the Dryad data repository at https://doi.org/10.5061/dryad.7pvmcvdv4 [42]. The data are provided in the electronic supplementary material [43].

## Footnotes

1 This study has shown that the difference in the amount of oxygen the fish used when accelerating to the critical swimming speed, and the amount of oxygen they would have used if they could achieve that speed aerobically, was actually consumed on top of the normal oxygen consumption during the first hour of recovery. This equity suggests oxidation of the lactate accumulated on the way to reaching the critical swimming speed and not toward its reuse as building blocks in gluconeogenesis, as in this case less lactate would have been available for oxidation, and less oxygen would have been consumed. It also suggests that the lactate was oxidized within the muscles, on top of sustaining the standard metabolic rate by other substrates. Having enough space to move, the energy released during the oxidation was probably dissipated into heat by the ‘restless behaviour’ of the fish.

2 Formally, parasite drag is the drag at zero lift and it mainly comprises of the friction between the body and the water; induced drag is the cost of generating hydrodynamic lift.

3 The first term in this equation is the potential energy of the swimmer; it has a negative sign because depth is the greatest at the nadir. The second term is the kinetic energy.

4 It can be interpreted as the speed that minimizes the cost of transport with zero metabolic rate.

5 Estimated hydrodynamic coefficients for *S.mokarran* were verified against wind-tunnel measurements reported in [3] and were similar.
